# Highlights from the 16th International Society for Computational Biology Student Council Symposium 2020

**DOI:** 10.12688/f1000research.53408.1

**Published:** 2021-06-04

**Authors:** Wim L. Cuypers, Handan Melike Dönertaş, Jasleen K. Grewal, Nazeefa Fatima, Chase Donnelly, Arvind Singh Mer, Spencer Krieger, Bart Cuypers, Farzana Rahman

**Affiliations:** 1 Adrem Data Lab, Department of Computer Science, University of Antwerp, Antwerp, Belgium; 2 Tropical Bacteriology Unit, Department of Clinical Sciences, Institute of Tropical Medicine, Antwerp, Belgium; 3 European Molecular Biology Laboratory, European Bioinformatics Institute EMBL‐EBI, Wellcome Trust Genome Campus, Cambridge, UK; 4 Canada’s Michael Smith Genome Sciences Centre, BC Cancer Research Institute, Vancouver, British Columbia, Canada; 5 ISCB Student Council, NA, Sweden; 6 Princess Margaret Cancer Centre, University Health Network, Toronto, Canada; 7 Department of Computer Science, University of Arizona, Tucson, Arizona, USA; 8 Molecular Parasitology Unit, Department of Biomedical Sciences, Institute of Tropical Medicine, Antwerp, Belgium; 9 School of Human and Life Sciences, Canterbury Christ Church University, Canterbury, UK

**Keywords:** Student Council Symposium, SCS, ISCB, ISCBSC, Virtual Seminar, Networking, ECR, conference, collaboration

## Abstract

In this meeting overview, we summarise the scientific program and organisation of the 16th International Society for Computational Biology Student Council Symposium in 2020 (ISCB SCS2020). This symposium was the first virtual edition in an uninterrupted series of symposia that has been going on for 15 years, aiming to unite computational biology students and early career researchers across the globe.

## Introduction

On 10-11 July 2020, early-career computational biologists from all over the world gathered virtually for the 16th International Society for Computational Biology (ISCB) Student Council Symposium (SCS). Preceding the Intelligent Systems for Molecular Biology (ISMB) conference, which is organised together with the European Conference on Computational Biology (ECCB) in Europe every two years, the SCS provides computational biology students and early career researchers attending these large conferences with an additional opportunity to connect with their peers, present their work, and exchange scientific experiences in a less-formal setting ( Francescatto et al., 2015). Therefore, the SCS is key in the mission of the ISCB Student Council (ISCBSC) to foster the development of the next generation of computational biologists, among a wide range of other initiatives, such as facilitating global student internships ( Anupama et al., 2018) and expanding the Regional Student Group (RSG) network ( Shome et al., 2016).

For the past 15 years, the ISCBSC has successfully organised the annual SCS in Europe, USA, and Canada ( Hassan et al., 2018). For the organising team, which is a different group every year, the SCS is a great opportunity to get familiar with the challenges and opportunities of organising an international meeting ( Parisi et al., 2019). Developing these organisational skills, including but not limited to time and project management, effective communication, and strong collaboration, is of crucial importance for academic and non-academic careers in science ( de Ridder et al., 2014).

The 16th edition of the SCS (hereafter SCS2020) was to be held in Montréal, Canada, as a satellite meeting of ISMB 2020. When the COVID-19 epidemic started to take pandemic proportions, the ISCB decided to gather virtually, allowing us to continue to share our computational biology research in these difficult times. Other key ISCBSC activities, including the European SCS ( Olguín-Orellana et al., 2021) and the Latin-American SCS ( Castillo-Vilcahuaman et al., 2020), were also organised online.

In this article, we discuss the highlights of SCS2020 and reflect on the challenges and benefits of organising a virtual event for a global community.

## Keynotes

The first keynote speaker, Professor Elana J. Fertig, from Johns Hopkins University, shared her research on matrix factorisation approaches to identify patterns in single-cell RNA sequence (sc-RNAseq) data. Combined with transfer learning, Dr. Fertig demonstrated the power of this methodology to identify axes of cellular identity. This technique can project the tumour sc-RNAseq data into latent axes and uncover cell types and cellular state transition events. Dr. Fertig also highlighted the need to develop biologically meaningful metrics for model evaluation since we often lack the ground truth in biology.

Professor Hamed S. Najafabadi, from McGill University, shared his interesting research journey that revolved around post-transcriptional regulation. In the talk, Dr. Najafabadi highlighted that an RNA-seq experiment is a snapshot of only the current state of a cell, and can contain crucial information on the level of RNA stability. By analysing large cancer datasets with a focus on transcript stability, Dr. Najafabadi and his team uncovered master regulators of cancer. He also emphasised the importance of re-analysing publicly available data, which often leads to new insights that can be evaluated in a lab.

## Oral presentations

A unique feature of the SCS is that all presentations cover very different biological topics. We welcomed more than 40 poster presenters in our virtual poster hall, which was accessible throughout the two-day event, but we asked presenters to be present for live Q&A via chat during a scheduled time slot. For the live program (mostly pre-recorded live Q&A), nine talks were selected for oral presentation, and six shorter poster presentations were selected for rapid poster flash talks. Here we summarise the work that was selected for an oral presentation.

### Day 1

Existing protein secondary structure prediction tools do not always leverage the increasing number of proteins with known secondary structure, instead performing homology search over proteins with no known structure. Spencer Krieger et al. formulated a new algorithmic approach for protein secondary structure prediction that combines both template- and non-template-based methods, resulting in a protein secondary structure prediction tool that surpasses state-of-the-art accuracy.

Folded proteins can contain regions of local energetic conflict, also known as frustration, which can be linked to protein function. Diego M. Luna presented his work on detecting evolutionary conserved frustration patterns that may be linked to specific functional adaptations in the globins protein superfamily.

Numerous biological and medical applications would benefit from a better understanding of gene expression regulation. Using deep learning, Jan Zrimec et al. demonstrated that mRNA abundances of different organisms can be predicted using information derived from both coding and non-coding DNA regulatory elements.

Designing peptides and proteins with a better affinity for a target *in silico* is a very promising approach to prioritise wet-lab experiments. For this purpose, Rodrigo Ochoa et al. developed PARCE, an open-source Protocol for Amino acid Refinement through Computational Evolution.

Soil microbiome diversity could be important to optimise farming strategies. Aneth Mwakilili et al. studied the patterns of diversity of soil microorganisms in monoculture plots versus plots where a maize push-pull farming strategy was adopted. They observed interesting trends, such as more uniform diversity in maize push-pull farming samples.

### Day 2

Different individuals metabolise certain drugs differently. Typing the underlying genetic factors is important for personalised treatment but can be challenging due to structural variations. David Twesigomwe et al. compared different allele-calling algorithms to study variation in the *CYP2D6* gene and proposed an ensemble method for accurate genotyping of *CYP2D6.*

Exploring the conformational diversity of proteins can be valuable in understanding their function. To study the conformational variety of spaces of proteins based on their equilibrium dynamics at room temperature, Tadeo Enrique Saldaño et al. presented a new iterative method that makes use of normal mode analysis according to protein elastic network models.

Long-read sequencing enables highly contiguous genome assemblies but is more prone to errors compared to short-read sequencing techniques. Janet Lorv et al. devised a method that uses existing assemblies or protein sequences to correct assemblies, resulting in less fragmented gene identifications and more accurate annotations, without the need for additional sequencing.

## The Black Women in Computational Biology network

The Black Women in Computational Biology (BWCB) network is a global initiative launched in 2020, aiming to serve as an intersectional and inclusive community for all women to share knowledge and thrive. We were honoured to invite Jenea Adams, the BWCB network founder and a PhD researcher at the University of Pennsylvania School of Medicine, who walked us through the BWCB philosophy and initiatives. She explained that it is important for her, as a first-generation student, not only to find mentors but also to connect with individuals who understand many intersections of being not only a woman in science but being a Black woman in science. The BWCB network started as a small online group and transformed into a global organisation that focuses on engaging current members and amplifying the voices of past and new members in the computational biology field.

The vision of how to further build the BWCB network, through a global citizenship perspective, involves several important components such as resource amplification and science communication. Science communication is a key element, Jenea Adams emphasised, along with intergenerational collaboration as it creates a space to share stories and journeys about navigating STEM as a Black woman. In addition to being a global organisation, the strength of the network lies in a group of senior academic and industry professionals that can help to build a foundation of mentorship framework. We encourage readers to visit the BCWB network website ( blackwomencompbio.org) to learn about how to be a part of it by contributing as a member or an ally to amplify and support important work being carried out by the BWCB network across the globe.

## Career panel

In addition to using the SCS platform to promote and highlight academic role models for the SCS community of young trainees, we organised an informative session on life beyond academia. The panel was moderated by Dr. Bruce Seet, founder of the Science to Business Network (S2BN) in Canada. It was run using Zoom webinar, which allowed a virtual separation of participants and organizers/panellists and enabled the audience to submit questions during the event easily. The panellists represented different career paths, including science consulting (Dr. Anne Mullin, Engagement Leader at Shift Health), entrepreneurship and industry science (Dr. Anthony Fejes, co-founder of HTuO BioSciences and ZymeWorks), and publishing (Dr. Rita Strack, Senior Editor at Nature Methods). The one-hour event was split between the initial introduction of the event and moderator by the event organizers (2-3 minutes), the moderator's outline of panel expectations (2-3 minutes), individual introductions by each panellist accompanied by an overview slide for online attendees (2-3 minutes), and a 40-minute Question and Answer (Q&A) session led by the moderator.

**Figure 1.**
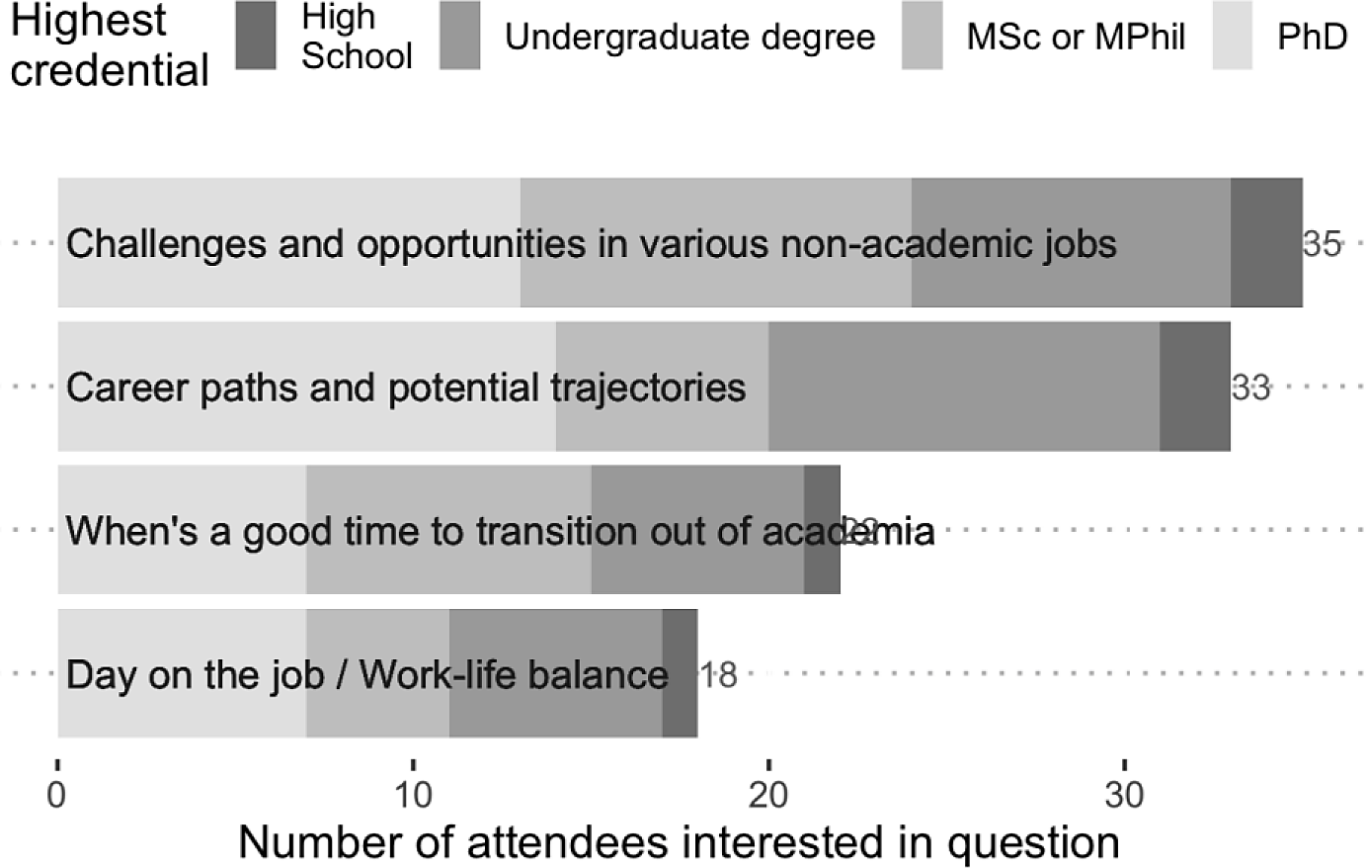
Ranking of potential questions that attendees wanted to see discussed by the career panel.

Three key departures were made from the standard panel in order to ensure a more representative, well-moderated online discussion. Firstly, participants were invited to respond to a question during the registration, which inquired what type of topics they would like to see discussed in the panel. The majority of respondents were interested in identifying alternative career paths, how they can be prepared for finding jobs outside of academia, and most importantly, the challenges and opportunities that go with the non-academic jobs ( Figure 1). An aggregate summary of these responses was provided to the panellists in advance to help them shape their introductions. Specific questions associated with each category were also picked by the organizers to help guide the initial Q&A. These strategies were extremely helpful in organizing the panel efficiently and in enabling the presenters to provide more directed, clear answers. Secondly, since the panellists and moderator did not have the chance of meeting each other in person, special effort was made to engage with the panellists virtually before the event. Lastly, we got an external panel moderator, Dr. Seet, who is more involved with the non-academic side of our field, to coordinate the discussion and to lead the Q&A. Dr. Seet brought in a track record of building community support and engagement strategies that facilitated transition out of academia, and this was evident in the Q&A.

## Social event

The SCS also included a social event over virtual platform, as a replacement for the yearly meetup that takes place at the end of the programme. An important part of student conferences is the ability to grow the young researchers' network and discuss interesting new projects that could not receive a time slot during the conference. This event was organised via the Zoom platform utilising breakout rooms to divide the group into more manageable sizes for discussion. The breakout groups were created based on different computational biology topics, and attendees were asked to join a room based on their area of interest. The ability of participants to freely travel between rooms allowed for people to join discussions on multiple topics and meet a more diverse group of people. The main takeaway for future online social events is that smaller groups allow for a better level of cooperation and discussion as larger groups tend to mute out the quieter personalities in the rooms and allow less time to get to know each individual in them.

## Fellowships

The event was free of charge for those registered for ISMB 2020, making SCS2020 accessible to all young scientists in our community. When not registered for ISMB 2020, a small fee was charged that could be waived when applying for a participation fellowship. Because of the generous sponsorship of Harvard Medical School, almost all requested participation fellowships were granted (N = 21).

For those participants in low and middle-income countries who do not have access to a stable internet connection, or lack the necessary equipment to join remotely in times of a pandemic, we provided the opportunity to apply for a remote participation fellowship. Three students received this fellowship to purchase materials that facilitate remote attendance (i.e. an internet data bundle or headset), one of whom was the best poster prize winner.

## Participants

The virtual format attracted a diverse audience from all around the world. There were more than 150 live attendants from 41 countries, spanning six continents. According to the voluntary survey completed by 64 participants, 62% of the attendants were already familiar with ISCBSC, but SCS2020 provided the first opportunity for 75% of the participants to attend the annual SCS. Indeed, 70% reported that they would not have been able to join SCS2020 if it was in person. This virtual event also provided a great opportunity to expand the SC network, as 70% showed an interest in joining ISCBSC or RSGs. Although it may not replace in-person meetings, there is no doubt that SCS2020 helped expand the SC network greatly.

## Awardees

Based on a rating by all delegates, prizes were awarded for the best oral presentations and posters. This was possible because of the sponsorship of Harvard Medical School Department of Biomedical Informatics.

In the best oral presentation category, the first place went to Spencer Krieger for his presentation titled: “Boosting the accuracy of protein secondary structure prediction through nearest neighbor search and method hybridization”. David Twesigomwe was awarded second place for his work titled: “Characterisation of CYP2D6 pharmacogenomic variation in african populations: an integrative bioinformatics approach”. The third place went to Janet Lorv for her work titled: “Gene de-fragmentation in long read assemblies using reference-based error correction”, and to Khawla Seddiki for her presentation titled: “Towards CNN representations for small mass spectrometry data classification: from transfer learning to cumulative learning”.

In the best poster presentation category, Rahagir Salekeen ranked first with his presentation titled: “ *In silico* study of human arachidonate 5-lipoxygenase inhibition potential of heritiera fomes extracted compounds”. Syed Muktadir Al Sium was awarded second place for his presentation titled: “AntiFam 6.0: Are we close to getting rid of spurious proteins in sequence databases?”. The third place went to Atilio Osvaldo Rausch for his presentation titled: “FrustratometeR: An R package to calculate energetic local frustration in proteins”, and Ina M. Deutschmann for her presentation titled: “Dynamic microbial association networks in the ocean”.

## Organisation and challenges

Most of the challenges associated with SCS2020 arose from the need to adapt the conference from an in-person to an online delivery format. The organising committees reacted quickly and moved to an online-only format for coordination and planning. Preliminary planning meetings occurred around 6 months prior to the conference through Zoom, where the main program agenda and key components were identified. Here the main challenge was finding suitable times where all members of the organising committee could be present (members were spread between the western coast of North America and western Europe).

Slack emerged as an effective medium for routine communication, whereby channels were set up to streamline efforts in ‘career panel’, ‘fellowships’, ‘finances’, ‘program’, and ‘social event’ components of the symposium. A challenge here was tracking non-Slack communication with external stakeholders (including the ISMB leadership and event attendees). To this end, internal Slack channels like ‘emails’, ‘outreach’, and ‘virtual’ were established to allow organizers to indicate whether certain external communications had already been addressed by them. Accompanied by monthly Zoom organisers' meetings, we found Slack to be a suitable format for coordinating the organisation of large-scale symposiums.

Main changes to the pre-existing format included offering remote participation awards and supplements in lieu of travel awards, soliciting pre-recorded talks from presenters, and the programming of the event over two days with a four hour ‘live’ program (featuring mainly pre-recorded talks with live Q&A) to optimise time zone overlap. Except for the social event, which was held as multiple breakout rooms in a Zoom meeting, research talks and auxiliary sessions (career panel, BWCB talk) were held over Zoom webinar. Background communications between organizers were directed to Discord channels. There was also a virtual poster hall that was accessible throughout the event, which hosted poster PDFs and pre-recorded flash talks, with links to a Google Group for discussion. Although many interesting discussions were started, it did not trigger the same level of engagement as a face-to-face poster session or coffee break.

## Conclusion

A high number of participants from all over the world joined to share their passion for computational biology, in times where the COVID-19 pandemic complicates every aspect of life, perhaps making this gathering even more necessary for our community. Thanks to the impressive keynote lectures, scientific talks, poster presentations, and the social event, everyone was able to refuel with exciting new research and ideas. The high interest in our career panel indicates that this community seeks support beyond the scientific topics and is eager to engage with science beyond established domains of research, but has limited knowledge of available opportunities and career paths beyond academia.

The adoption of an online-only platform came with its challenges. First, casual conversations that would otherwise arise organically when in person are difficult to replace online. Second, the generation and consolidation of recorded talks is convenient once in place but requires additional expertise with video editing and managing online media. Last, catering to a global audience means that events cannot always be made accessible to participants in different time zones while maintaining the same level of engagement. To address these challenges, we provided participants with ‘tips & tricks’ for recording an effective presentation for virtual sharing, made talks and posters available online with the option to comment, organised social events, and opted to spread the event over two days with optimal time-zone overlap. Other workarounds could include building moderated breakout rooms, or using virtual interaction spaces like gather.town (not available at time of conference). Overall, we believe that overcoming these challenges should be prioritised by symposium organisers, as we learned that these investments are key to optimise participant engagement.

During SCS2020, we also realised that it is not a self-evident process for all scientists to “create a seat at the table”. A higher representation of scientists from the low- and middle-income regions for this virtual event compared to the previous face-to-face symposia, combined with the fact that 70% of our survey respondent participants indicated that they would have been unable to attend SCS2020 in person, highlights the financial and geographical inequity implicit in on-site international conferences. We feel even more strengthened as a community to proceed with our mission to facilitate connections between early-career computational biology researchers around the globe. We therefore suggest that online conferences should be considered not as a temporary replacement but a permanent complement to in-person events.
